# Vegetation changes and land surface feedbacks drive shifts in local temperatures over Central Asia

**DOI:** 10.1038/s41598-017-03432-2

**Published:** 2017-06-12

**Authors:** Xiuliang Yuan, Wenfeng Wang, Junjie Cui, Fanhao Meng, Alishir Kurban, Philippe De Maeyer

**Affiliations:** 1 State Key Laboratory of Desert and Oasis Ecology, Xinjiang Institute of Ecology and Geography, Chinese Academy of Sciences, Urumqi, 830011 China; 2 University of Chinese Academy of Sciences, Beijing, 100049 China; 3 Department of Geography, Ghent University, Ghent, 9000 Belgium; 4 Sino-Belgian Joint Laboratory of Geo-information, Urumqi, 830011 China and Ghent 9000 Belgium

## Abstract

Vegetation changes play a vital role in modifying local temperatures although, until now, the climate feedback effects of vegetation changes are still poorly known and large uncertainties exist, especially over Central Asia. In this study, using remote sensing and re-analysis of existing data, we evaluated the impact of vegetation changes on local temperatures. Our results indicate that vegetation changes have a significant unidirectional causality relationship with regard to local temperature changes. We found that vegetation greening over Central Asia as a whole induced a cooling effect on the local temperatures. We also found that evapotranspiration (ET) exhibits greater sensitivity to the increases of the Normalized Difference Vegetation Index (NDVI) as compared to albedo in arid/semi-arid/semi-humid regions, potentially leading to a cooling effect. However, in humid regions, albedo warming completely surpasses ET cooling, causing a pronounced warming. Our findings suggest that using appropriate strategies to protect vulnerable dryland ecosystems from degradation, should lead to future benefits related to greening ecosystems and mitigation for rising temperatures.

## Introduction

Recent studies indicate that the Earth is experiencing a profound greening trend as a result of elevated CO_2_ fertilization, climate change and land cover change^1, 2^. Coincident with vegetation greening, evapotranspiration (ET) has been increasing over the past three decades at a rate of ~0.88 mm year^−1^, and more than half of the solar energy absorbed by land surfaces has been dissipated^3, 4^. Today, the Northern Hemisphere would be 15–25 °C warmer if the terrestrial ET was zero^5^, implying that an increase in ET associated with vegetation greening has a strong influence on locally cooling temperatures. In contrast, vegetation greening can also warm local temperatures by reducing albedo^6^. Since the presence of forests can warm local temperatures by 12 °C in April and by 5 °C in July in Arctic and sub-Arctic regions, the climate effects of these albedo changes are substantial^7^. Therefore, understanding the feedbacks of vegetation changes and their capacity to influence local temperatures is helpful to further identify global warming and the development of appropriate strategies required to protect ecosystems.

The potential effects of vegetation changes on local temperatures due to alterations in energy storage and transfer have been investigated for a long period of time. Research obtained from the afforestation programme in northern China indicates that increases in vegetation coverage have a cooling effect and that the range of the identified temperatures could possibly be mediated based on the Granger causality test^8^. Another study indicates that due to increased nighttime temperatures, in relation to decreased daytime temperatures, afforestation in China has resulted in a net warming within the arid and semiarid regions^9^. Agricultural practices can also have an influence on temperatures. A recent study suggested that agricultural intensification increased the potential for ET, leading to cooler temperatures^10^. Negative feedbacks on growing season temperature due to increases in vegetation productivity have also been observed on the Tibetan Plateau^11^. Based on land surface model simulations, there was found that albedo changes have a positive feedback on climate change, especially in high latitudes. Positive forcing induced by decreases in albedo were determined to be capable of offsetting the negative forcing that had been anticipated from carbon sequestration in many boreal forest areas^12, 13^. However, since the climate implications of ecosystem feedbacks tend to be local, conclusions from the studies mentioned above are divergent. Some are even contradictory. Given the spatial heterogeneity of the natural environment, the net forcing of vegetation changes in various geographical locations remains uncertain and difficult to determine. Thus, a comprehensive understanding of the feedbacks of vegetation changes on local temperatures for various geographic categories is required.

Central Asia is located deep inside the Eurasian continent and includes Kazakhstan, Kyrgyzstan, Tajikistan, Turkmenistan, Uzbekistan, and the Xinjiang Province of China^14, 15^. The area contains a large fraction of dryland (75%) which consists of desert, shrub, and other water stressed plant communities. The area also contains a large fraction of relatively humid regions (25%) such as needleleaf forests, broadleaf forests, and croplands, largely near mountainous areas. In recent decades, Central Asia has experienced a vegetation greening, as well as rapidly increasing temperatures^16^. The annual mean temperatures in the region have been increasing at an average rate of 0.39 °C decade^−1^ from 1979 to 2011; this is larger than the average rate for global land areas (i.e., 0.27–0.31 °C decade^−1^ from 1979 to 2005)^14^. Horton, *et al*.^17^ found that these thermodynamic contributions are largely independent of atmospheric circulation and potentially related to surface processes such as land cover change, factors that allowed us to investigate the climate effects of vegetation change feedbacks.

The major questions addressed in this study were the following:

1. How has vegetation changed during the period from 2000 to 2014?
2. Do vegetation changes influence local temperatures significantly?
3. If so, how do these influences vary in different geographical regions?
4. How much do ET and albedo, associated with vegetation changes, contribute to local temperatures changes?

## Results

**Figure 1:**
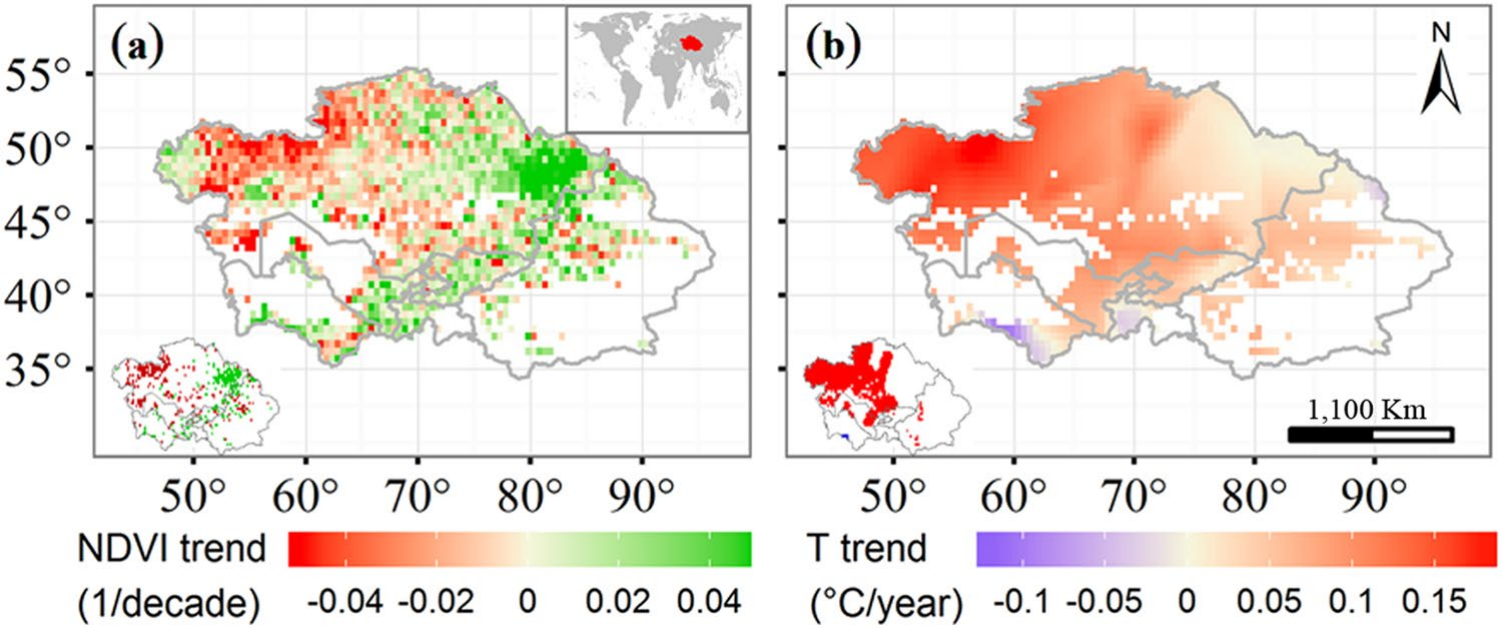
Spatial linear trends for the growing season Normalized Difference Vegetation Index (NDVI) (**a**) and temperature (T) (**b**) in Central Asia from 2000–2014. The insets indicate pixels that are statistically significant at *p* < 0.05. Maps were generated by using free software R (R Core Team (2015). R: A language and environment for statistical computing. R Foundation for Statistical Computing, Vienna, Austria. URL: https://www.R-project.org/).

The Normalized Difference Vegetation Index (NDVI), derived from the contrast between the red and near-infrared reflection of solar radiation, has commonly been used to reflect the growth conditions of the vegetation activity (photosynthesis and vegetation coverage)^18^. Due to this fact, we first investigated the spatial distribution of long-term changes in growing season NDVI across Central Asia. Large areas of Central Asia (55%) have undergone greening from 2000 to 2014 (Fig. 1a), with 19% of the positive pixels being significant (*p* < 0.05). Approximately 45% of Central Asia has undergone a decrease in NDVI, with 13% of the negative pixels being significant, largely within the northwestern portion of Central Asia. Nearly the entire area of Central Asia has experienced warming, with 38% of pixels being significant (Fig. 1b).

**Figure 2:**
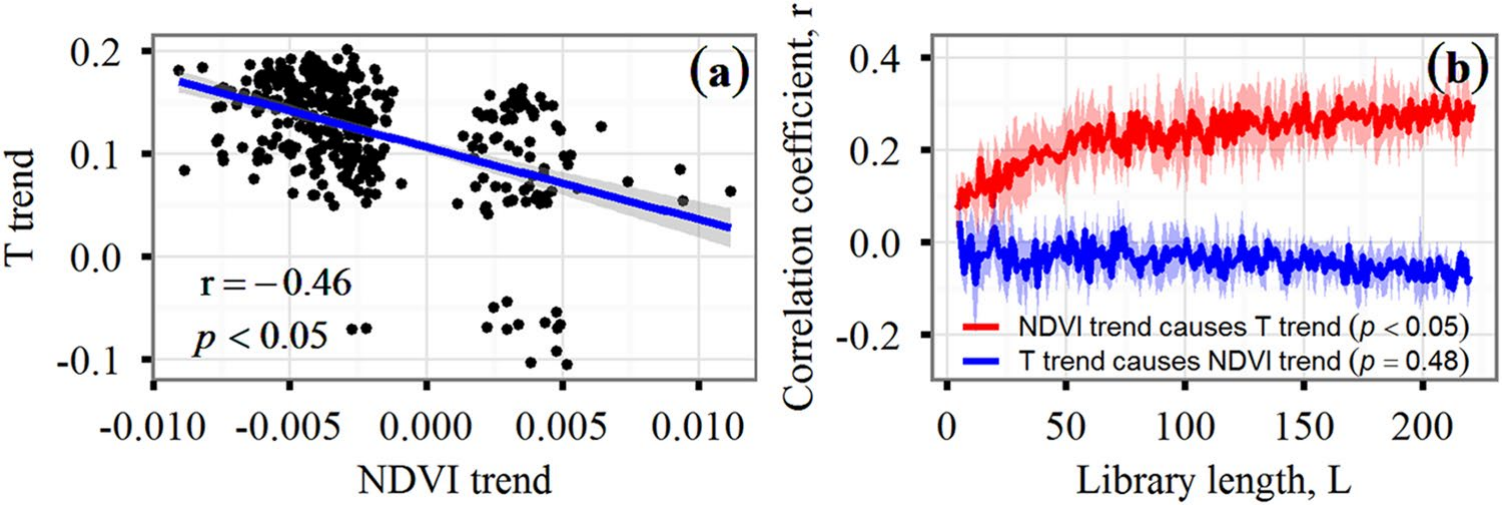
The linear regression (**a**) and convergent cross mapping (CCM) causality (**b**) relationship between the linear trend of the NDVI (NDVI_trend_) and temperature (T_trend_). Shadows on either side of the CCM curves represent ±standard error. The library length, L, is the number of observations (pixels with significant trend (*p* < 0.05) for NDVI or temperature). The NDVI_trend_-reconstructed T_trend_ gradually converges to a large positive correlation coefficient (r = 0.35) whereas the T_trend_-reconstructed NDVI_trend_ displays flat curves with a low correlation coefficient, suggesting that the NDVI_trend_ has a significant unidirectional causality relationship with the T_trend_.

For the purpose of our experiment, we hypothesized that vegetation greening influences local temperatures due to land surface feedbacks. Further, we postulated that an increase in temperatures occurs due to a reduction in surface albedo and a decrease in temperatures by increase of ET. To test this hypothesis, a regression relationship was first employed for establishing the strength of the association between the NDVI trend (NDVI_trend_) and the temperature trend (T_trend_) so that we could form an initial description of potentially relevant relationships. A negative correlation was found (r = −0.46, *p* < 0.05) (Fig. 2a), implying that a reduction in temperature with rising ET dominated increases in temperature due to a reduction in surface albedo. However, the regression relationship does not allow us to formulate a cause-and-effect relationship. To address this issue, convergent cross mapping (CCM) was used to identify causality and the direction of causality in the presence of system feedbacks. Our results indicated that unidirectional causality for the NDVI_trend_ provided stronger control over the T_trend_ (*p* < 0.05) (Fig. 2b) for the “NDVI_trend_ causes T_trend_” than for the “T_trend_ causes NDVI_trend_”. Furthermore, CCM did not show significant causality for the “T_trend_ causes NDVI_trend_” (*p* = 0.48).

**Figure 3:**
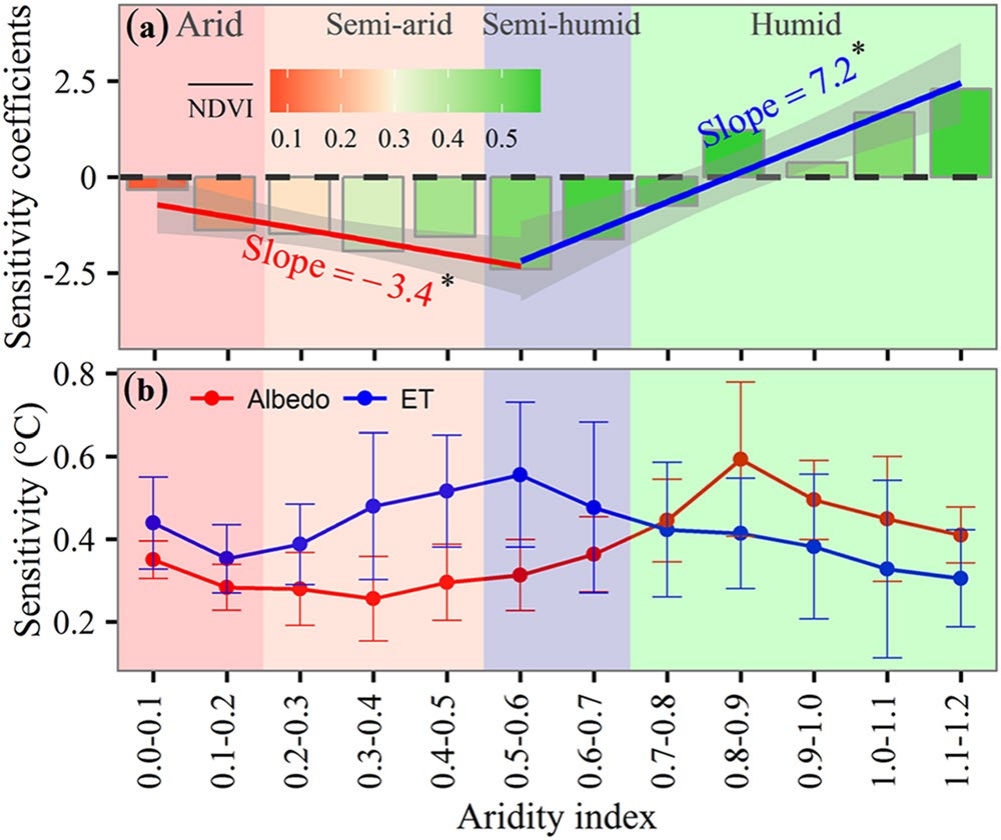
Sensitivity of the temperature trend to NDVI trend (**a**) and temperature sensitivity of evapotranspiration (ET)/albedo to the NDVI changes (every 0.1 increment) across hydroclimatic regimes (**b**). In (**a)**, the color for each bar indicates the mean growing season NDVI. Hydroclimatic regimes are represented by the aridity index. Pixel values were averaged using a bin (0.1) for the aridity index. The value for each bar was calculated using the mean sensitivities for each bin, with statistical significance at *p* < 0.05. The solid line represents the least squares regression. The labeled slopes indicate change in sensitivity of the temperature trend to NDVI trend corresponding to per 0.1 increase in aridity index, and that with an asterisk are significant (*p* < 0.05).

To further explore the dependency of the T_trend_ on the NDVI_trend_ at any given location, we calculated the T_trend_ sensitivity coefficients for NDVI_trend_ across Central Asia and grouped the results into four aridity categories (arid, semi-arid, semi-humid, and humid). A notable downward unimodal pattern for coefficient distribution was observed, with peak values occurring within the semi-humid region (0.5 < AI < 0.6) where the mean growing season for the NDVI was approximately 0.4 (Fig. 3a). In the arid, semi-arid, and semi-humid categories, the data displayed negative sensitivity coefficients and the values decreased significantly. The sensitivity coefficients rapidly increased from a minimum value and changed into a positive for humidity, reflecting different sensitivities for the T_trend_ in relation to the NDVI_trend_ over Central Asia across hydroclimatic variations.

**Figure 4:**
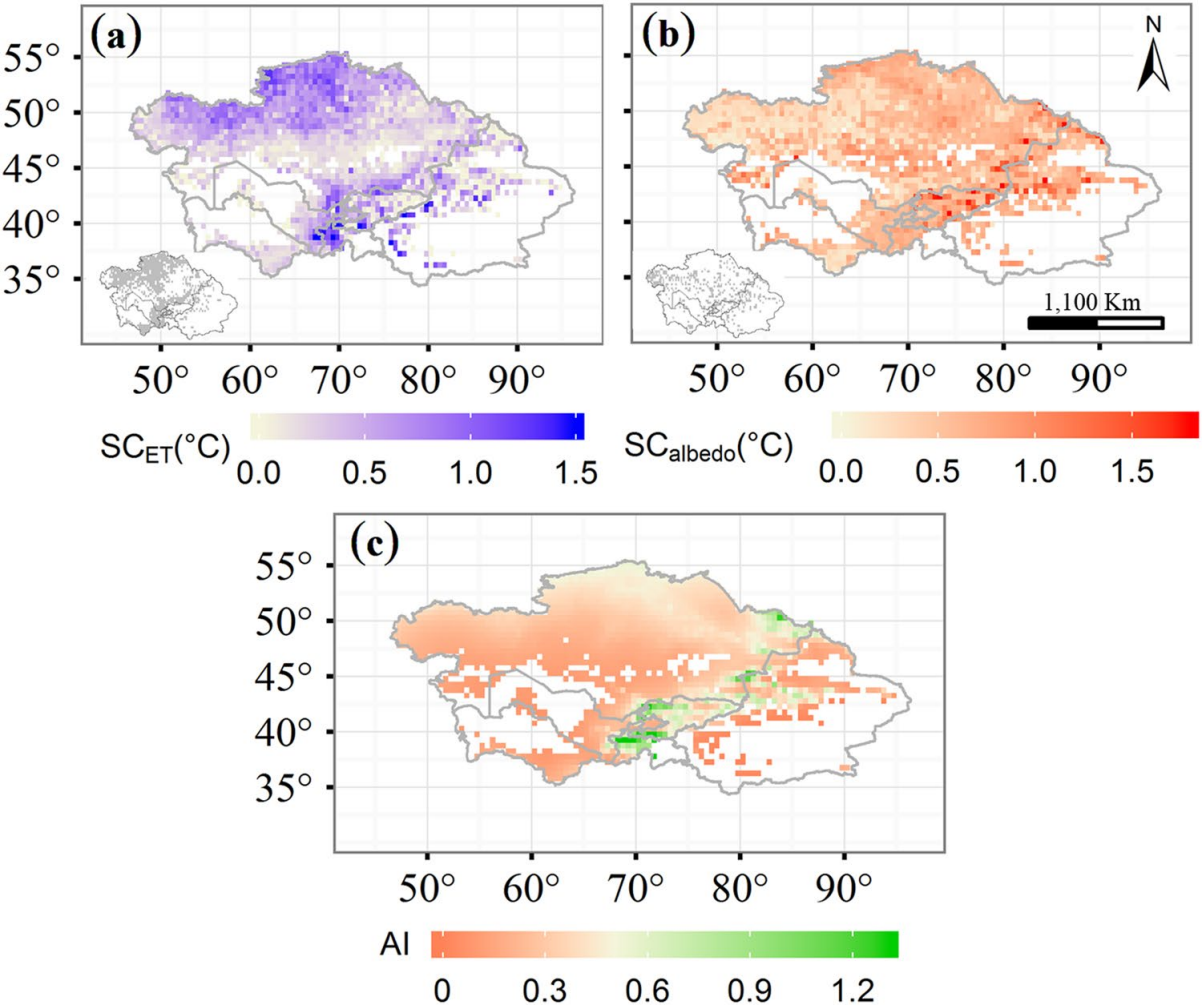
The spatial distribution of the **s**ensitivity of evapotranspiration (ET) (**a**), albedo (**b**) to the NDVI changes (every 0.1 increment), and the spatial distribution of the aridity index (**c**). The map was generated by using free software R (R Core Team (2015). R: A language and environment for statistical computing. R Foundation for Statistical Computing, Vienna, Austria. URL: https://www.R-project.org/).

Vegetation greenness increases the amount of absorbed solar radiation at the land’s surface and this extra energy is largely dissipated by ET associated with surface cooling. To quantify the relationship between vegetation greenness and temperature, we performed a linear regression analysis with ET or land surface albedo set as the dependent variable and the NDVI was set as the independent variable. Our results indicated that the regression slopes were all positive for ET and negative for albedo. Converting the unit for the regression slopes to temperature according to Shen, *et al*.^11^, indicates that the ET-induced cooling effect was larger in the eastern and northwestern Central Asia, largely humid areas (Fig. 4). In the northwestern region, which is largely arid, the ET-induced cooling effect was lower. In a similar manner, albedo-induced warming effects were larger for eastern regions than for southwestern regions. In arid, semi-arid, and semi-humid regions, the ET-induced cooling effect due to vegetation greening was larger than the warming effect induced by albedo; it was the inverse in humid regions (Fig. 3b). Extra radiation values for absorption due to vegetation greenness were approximately 0.32 ± 0.05 °C, 0.28 ± 0.09 °C, 0.34 ± 0.09 °C, and 0.47 ± 0.12  °C, and those for dissipation due to ET were approximately 0.40 ± 0.11 °C, 0.46 ± 0.14 °C, 0.52 ± 0.19 °C, and 0.37 ± 0.16 °C in response to a NDVI increment of 0.1 for arid, semi-arid, semi-humid, and humid regions, respectively (Table 1). Extra energy absorbed by albedo was less than the extra energy dissipated by ET, resulting in a cooling effect for arid, semi-arid, and semi-humid regions. On the contrary, the amount of energy absorbed by albedo exceeded that dissipated by ET, resulting in a warming effect for humid regions.

**Table 1:** The sensitivity (mean $\pm$ standard deviation) of evapotranspiration (ET)/albedo to NDVI changes (every 0.1 increment).

Sensitivity (°C)	Arid	Semi-arid	Semi-humid	Humid
ET	0.40 (±0.11)	0.46 (±0.14)	0.52 (±0.19)	0.37 (±0.16)
Albedo	0.32 (±0.05)	0.28 (±0.09)	0.34 (±0.09)	0.47 (±0.12)
Difference	0.08* (±0.07)	0.18* (±0.11)	0.18* (±0.11)	−0.1* (±0.13)

“*”indicates a significant difference at the confidence level of *p* < 0.05.

## Discussion

Changes in vegetation greenness reported at regional and continental scales have been associated with the effects of CO_2_ fertilization, climate change and land use^19, 20^. However, the feedbacks of vegetation changes on climate remain poorly known and uncertain, especially within Central Asia, which is sensitive and susceptible to climate change and environmental degradation^21^ and consists of large arid and semi-arid regions (75%). A previous study indicated that transpiration within arid regions ranges from 70–80% of ET^22^. As a result, vegetation greenness increases the potential for ET that can modify temperatures through alterations in surface energy. In this work, we investigated the impacts of the NDVI_trend_ on the T_trend_ and found that the NDVI_trend_ had a significant negative influence on the T_trend_ over Central Asia. The result indicates the dominance of ET-induced cooling over albedo-induced warming for cases of increased vegetation greenness. Our results are consistent with previous studies for middle latitudes within the Tibetan Plateau^11^ and the US Midwest^10^, but contradict studies for Artic regions^7, 23^. Here, we note that Arctic temperatures are relatively lower than those within middle latitude regions, resulting in lower ET.

In addition to a statistical correlation, the test of CCM causality also indicated that the NDVI_trend_ can cause the T_trend_ to be significant, but that the T_trend_ cannot significantly influence the NDVI_trend_. Since a previous study has reported that vegetation greenness increases water vapor within water-limited regions^8^, unidirectional causality is not surprising and may be one of the main factors influencing air temperatures. Such a hypothesis is reasonable for our study area given that increased water vapor caused by increased vegetation dissipated more energy than that absorbed by decreasing albedo within arid, semi-arid, and semi-humid regions. Since the warming trend increased within Central Asia, increasing temperatures may have caused a larger water deficit due to ET losses, thereby increasing plant water stress and desiccation and impacting vegetation growth through photosynthesis. One would also expect that the warming trend could lead to the decrease of NDVI. However, due to long-term limits induced by extreme aridity and high temperatures, vegetation in dry land regions has evolved into rich and deep root systems and high root/shoot ratios^24^, which could mitigate the effect of increased temperatures on the NDVI. Such a finding further supports our suggestion that the T_trend_ cannot significantly cause the NDVI_trend_. Additionally, after the collapse of the Union of Soviet Socialist Republics (USSR), the Central Asian countries experienced large changes in land-use followed by socio-economic disturbances (e.g. policy changes and economic crises)^25^. Millions of hectares of farmland were abandoned in northern Central Asia^26^, which likely contributed to the decline of vegetation greenness in these regions. However, to alleviate environment damage resulting from the over-extension of irrigated areas, actions have been taken to rehabilitate abandoned croplands and to plow rain-fed crops in southern and eastern Central Asia^25^. Therefore, precipitation, and not temperatures, has been determined to be highly and positively correlated with vegetation greenness in these areas^16^.

Given the spatial heterogeneity of the natural environment, determining net forcing within various geographical locations is insufficient because the feedback mechanisms of vegetation changes and its relationship to local temperatures are complicated. However, in contrast to the examined arid, semi-arid, and semi-humid regions, our results indicate that in humid regions the increased NDVI (13% of Central Asia) amplifies local warming. Here, it should be noted that humid regions generally have a higher soil moisture, leading to more potential heat storage during the day, more heating during the night, and the offset of ET-induced cooling during the day^9^. Additionally, humid regions have higher vegetation coverage, as represented by the mean growing season NDVI in Fig. 3a, and have a more stable stratification and, thus, less turbulence, known to remove water vapor^23^, as compared to dry land with lower vegetation coverage. Furthermore, precipitation occurs more frequently in humid regions within Central Asia^27^ and is associated with more cloud formation that may result in more downward longwave radiation received from the atmosphere and less outgoing longwave radiation than that for dry lands^9^.

The unimodal patterns of sensitivities for the T_trend_ to the NDVI_trend_, across the aridity gradient, are important and unique findings in this study. The result implies that feedbacks of vegetation change for local temperature changes follow hydroclimatic variations, from negative to positive. Within the scientific literature, similar results have been verified for other regions. For example, vegetation greenness within the arid/semi-arid zones of northern China has a negative influence on temperature^8^. Within the drylands of the mid-western US, increased cropland intensification is associated with cooling^10^. For the Tibetan Plateau, with large semi-arid/semi-humid areas, increased vegetation productivity caused a negative feedback on temperature^11^. However, increasing vegetation activity is estimated to warm local climates within the arctic under relatively humid environments^23^. A recent report has projected an accelerated dryland expansion, and dryland areas have been projected to increase by 11–23% by the end of the century^28^. Our results indicate that vegetation greenness will mitigate increasing temperature trends in these areas. Therefore, protecting native vegetation from degradation and afforestation will be beneficial to control global warming effects.

We used MODIS products (ET and Albedo) for assessing the contributions of each component on temperature variations. However, remote sensing data have limitations including instantaneous signals, optical-infrared constraints over cloudy regions, and transducer sensitivity^29, 30^. Nevertheless, in this work, we have provided important insight regarding the feedback of vegetation greenness on temperature changes. To explore the relevant physical mechanisms, long-term observations should be undertaken.

## Conclusion

Since the beginning of 21^st^ century, Central Asia has undergone significant vegetation changes. In this work, we conclude that vegetation greenness can cause significant temperature changes and that greening has a cooling impact on local warming over the entire Central Asia region. However, feedbacks for vegetation greenness in regards to local temperatures differ for different hydroclimatic geographical zones. A contrasting response for vegetation greenness on temperature changes exists between the arid/semi-arid/semi-humid and the humid regions and can be attributed to differences in the magnitudes of ET-induced cooling and albedo-induced warming. In arid/semi-arid/semi-humid regions, ET-induced cooling was found to dominate albedo-induced warming, while the result was the inverse for humid regions. Due to nonlinear interactions, temperature changes are therefore the result of multiple factors in complicated systems. For this study, we largely focused on ET and albedo changes as a result of vegetation greenness, two of the major factors influencing local temperatures within our study area that is located deep inside the Eurasian continent. More comprehensive, localized and in-depth analyses are required in the future.

## Methods

### Datasets

The collection 5 MODerate resolution Imaging Spectroradiometer (MODIS) NDVI dataset, at a spatial resolution of 0.05° and with a 16-day time-step from 2000–2014, was used to characterize vegetation changes. The dataset is well known for its high quality and has been widely used in previous studies^9, 11^. The monthly mean temperature dataset CRU 3.24, a climate dataset with a spatial resolution of 0.5° spanning 2000 to 2014, was obtained from the Climatic Research Unit (CRU) at the University of East Anglia^31^. We used global validated MODIS products for ET and albedo. Monthly MODIS ET data (MOD16), from 2000 to 2014, with a spatial resolution of 0.05°, have been previously examined using both global and local eddy flux towers, and have provided critical information regarding global terrestrial water and energy cycles and environmental changes. From 2000 to 2014, MODIS albedo (MCD43B3) products had a temporal resolution of 8 days and a spatial resolution of 1 km^32^. White sky albedo was used for our study. To understand the dependency of climate feedback effects on vegetation changes across hydroclimatic regimes, the aridity index (AI) was used to define geographic categories (i.e., ‘arid’: 0.03 < AI < 0.2, ‘semi-arid’: 0.2 < AI < 0.5, ‘semi-humid’: 0.5 < AI < 0.65, ‘humid’: AI > 0.65) according to the United Nations Environment Programme^33^; and was calculated using the following equation: AI = MAP/PET (MAP: Mean Annual Precipitation; MAE: Mean Annual Potential ET).

### Data processing

The original MODIS data were filtered based on the Quality Control (QC) layers. After filtering out low-quality pixels, gaps were filled using linear interpolation through two adjacent data points. Time series with more than two consecutive gaps were excluded from further analyses. Over the entire study area and over a 15 year time period, a total of 1.6% and 2.3% of all of the data were gap-filled for EVI and albedo data, respectively. To match CRU data, all of the data were interpolated to 0.5°. Annual mean values for MODIS NDVI pixels less than 0.1 were ignored so that non-vegetated regions were eliminated^8^. In order to eliminate the potential influence of autocorrelation in the NDVI and temperature time series on trend-detection, the *t-test* was first applied to test the significance of the lag 1 autocorrelation coefficient r1. If the autocorrelation exist under the confidence level, *p* < 0.05, the lag 1 autoregressive (AR(1)) process by pre-whitening was applied to remove autocorrelation from the time series of the NDVI and temperature according to Yue and Wang^34^, using the following equation

1 $$X{\text{'}}_{t}\,=\,{X}_{t}\,-\,r1\,\times \,{X}_{t-1}$$

where $r1$ is the lag 1 autocorrelation coefficient, *X* is the data value (i.e., NDVI, T), and t is the length of the time series.

### Analysis

Firstly, a linear regression analysis was used for establishing the strength of association between the linear trend of NDVI and temperature, in order to help form an initial description of the potential relationships. To quantify the feedback of vegetation changes on ET and albedo during the growing season (April through October), we performed a linear regression analysis in which the NDVI was set as the independent variable and ET or albedo was set as the dependent variable for each pixel. The slope of the regression function was considered to be the sensitivity coefficient. Since MODIS data shared model covariates across products, potentially resulting in autocorrelation in linear regression models, the *Breusch-Godfrey test* ^35^ was used to determine the significance of autocorrelation. If autocorrelation existed under the confidence level, *p < *0.05, the generalized least squares (GLS) method was used to eliminate autocorrelation according to the following equation of Hansen^36^:

2 $${y}_{t}\,-\,\rho {y}_{t-1}\,=\,{\beta }_{0}\,(1\,-\,\rho )\,+\,{\beta }_{1}\,({x}_{t}\,-\,\rho {x}_{t-1})$$

where ${y}_{t}$, and ${x}_{t}$ are the dependent and independent variables, respectively, and ${\beta }_{0}$, ${\beta }_{1}$, and $\rho$ are the intercept, slope, and autocorrelation coefficient, respectively, of an estimated linear regression equation. The GLS analysis was implemented by using the free *nlme* package in the R language environment^37^.

**Figure 5:**
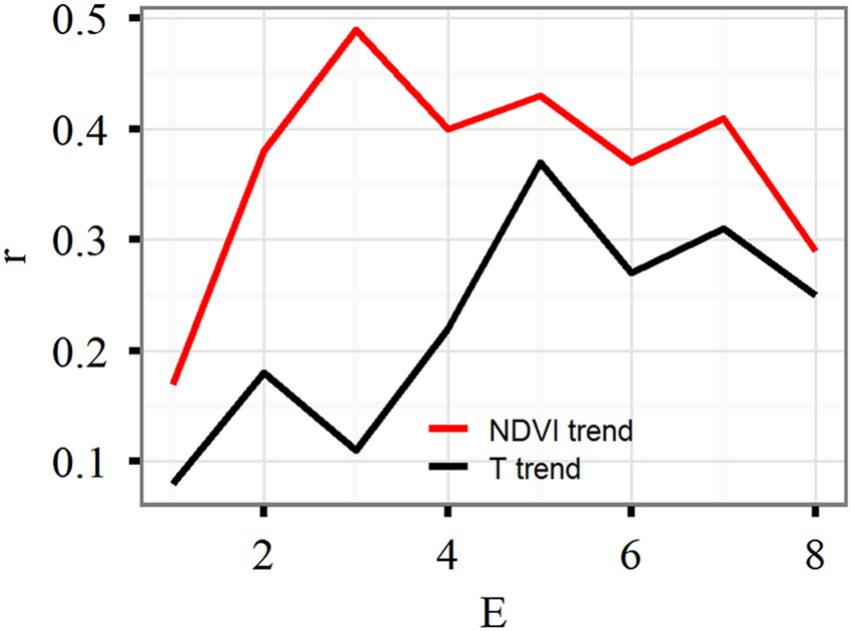
Prediction skill (correlation coefficient between actual and predicted values, r) as a function of the embedding dimension (E) using the simplex projection method of Sugihara and May^40^.

The convergent cross mapping (CCM) method^38^ was used to detect causal relationships between the NDVI trend and the temperature trend. The CCM is based on the theorem proven by Takens^39^ which states that the essential information of a multidimensional dynamical system is retained within the time series of any single variable of that system and that the extent of historical registrations in one variable can consistently approximate a second variable. If variable x is influenced by y, then causality is established if a causal variable x can be recovered from a variable y. The CCM method checks for causation by measuring the potential correlation between predictions and actual observations. Simplex projection^40^ was applied in order to reconstruct the dynamics of one process from another. Reconstruction was performed using an embedding dimension, E, that should have the highest skill for prediction. In our analysis, E was 3 for the NDVI trend and 5 for the temperature trend (Fig. 5). In general, causal relationships were suggested if convergence was present and if the Pearson’s correlation was greater than zero, while non-causal relationships were illustrated if a flat curve was present. The variable with the highest Pearson correlation coefficient at the point of convergence indicates the strongest controlling variable. Detailed information can be obtained from a short instructional animation: https://www.youtube.com/playlist?list=PL-SSmlAMhY3bnogGTe2tf7hpWpl508pZZ. The CCM analysis was implemented by using the free multispatial CCM package within the R language environment^41^.
